# Food Insecurity Is Associated with Mental–Physical Comorbidities among U.S. Adults: NHANES 2013 to 2016

**DOI:** 10.3390/ijerph19031672

**Published:** 2022-02-01

**Authors:** LaToya J. O’Neal, Ara Jo, Lisa Scarton, Marino A. Bruce

**Affiliations:** 1 Department of Family, Youth and Community Sciences, Institute of Food and Agricultural Sciences, College of Agricultural and Life Sciences, University of Florida, Gainesville, FL 32611, USA; 2 Department of Health Services Research, Management and Policy, College of Public Health and Health Professions, University of Florida, Gainesville, FL 32611, USA; ara13j@phhp.ufl.edu; 3 Department of Family, Community and Health Systems Science, College of Nursing, University of Florida, Gainesville, FL 32611, USA; lscarton@ufl.edu; 4 Department of Behavioral and Social Sciences, College of Medicine, University of Houston, Houston, TX 77004, USA; mabruce@central.uh.edu

**Keywords:** food insecurity, comorbidity, depression, chronic conditions, NHANES, cardiometabolic conditions

## Abstract

The co-occurrence of mental and physical conditions has increased significantly during the last decade. However, research examining the influence of social factors such as food insecurity is limited. The purpose of this study was to examine the association between food insecurity and mental–physical comorbidity status among U.S. adults. Data for this analysis were drawn from the National Health and Nutrition Examination Survey (NHANES) for the years 2013–2016. Respondents ages 18 and older who reported at least one of three chronic conditions (i.e., type 2 diabetes mellitus, hypertension, and hyperlipidemia) and responded to a nine-item depression scale were included in the analytic sample. The prevalence of food insecurity among those with depression and a cardiometabolic condition was 34% compared to 13% among those with a cardiometabolic condition only. Findings from multinomial logistic regression models indicated that food insecurity was associated with higher risk of mental–physical comorbidity (OR: 3.6, 95% CI: 2.26–5.76). Respondents reporting poor diet and poor self-reported health had higher odds of comorbid depression and cardiometabolic conditions. Female respondents had increased odds of comorbid depression and cardiometabolic conditions. Food insecurity is associated with co-occurring depression and cardiometabolic disease and may have implications for disease management.

## 1. Introduction

Food insecurity is a critical risk factor for poor health outcomes, especially those related to dietary intake [1,2]. The significant association between household food insecurity and cardiometabolic comorbidity is a growing health concern, as the prevalence of U.S. adults living with multiple chronic conditions has increased significantly over the last two decades [3,4]. Depression is one of the chronic conditions that co-occurs with a number of cardiometabolic diseases (type 2 diabetes mellitus (T2DM), hypertension, and hyperlipidemia) linked to food insecurity. Recent data indicate that the proportion of individuals with depression and T2DM is 15.4% [5], that the prevalence of depression with hypertension is 26.8% [6], and that depression with hyperlipidemia is 29.7% among some populations [7]. The prevalence of mental–physical comorbidities is troubling given that individuals who experience depression along with chronic cardiometabolic conditions tend to have higher morbidity, mortality, and healthcare expenses [8,9,10] than individuals with a single chronic disease. Depression has been shown to negatively impact adherence and self-activation, which are essential for managing T2DM, heart disease, and hypertension [11,12,13,14].

Social factors such as food insecurity have been found to impact health [1,2,15,16], yet, few studies have examined specific associations between food insecurity, depression, and chronic disease. Further, none have examined the relationship between food insecurity and comorbid depression and cardiometabolic conditions among a nationally representative sample. Identifying risk factors associated with co-occurring mental and physical conditions requires attention, as this type of comorbidity can accelerate disease progression and contribute to the development of complications and more severe diseases (e.g., chronic kidney disease, cardiovascular disease) that exacerbate the costs and burdens to individuals, families, and communities [17,18,19,20,21].

The purpose of this study is to (1) characterize chronic cardiometabolic conditions and their co-occurrence with depression in a nationally representative sample of the U.S. population and (2) assess the salience of food insecurity on chronic cardiometabolic conditions and mental–physical comorbidities among adults in the U.S. We expect food insecurity to be associated with a greater likelihood of mental–physical comorbidities.

## 2. Materials and Methods

### 2.1. Sample

Data for this analysis were drawn from the National Health and Nutrition Examination Survey (NHANES) for the years 2013–2016. The NHANES uses multistage probability complex sample design to establish a nationally representative sample of civilian, noninstitutionalized persons in the U.S. Data for NHANES were collected via household survey and physical examination and included sociodemographic and clinical information. The analytic sample for this study was limited to adults ages 18 and older who reported medical conditions of three chronic diseases (i.e., T2DM, hypertension, and hyperlipidemia) and responded to a nine-item depression scale. Participants who have missing values on one of the targeted medical conditions (n = 3029) and pregnant women (n = 85) were excluded. The analytic sample for this study consisted of 8948 participants. This study was approved as exempt by the Institutional Review Board at the University of Florida.

### 2.2. Study Measures

#### 2.2.1. Comorbidity Status

Mental–physical comorbidity status was the primary outcome for the study. This three-category measure was derived to capture whether respondents had no chronic conditions, at least one chronic cardiometabolic condition (hypertension, T2DM, or hyperlipidemia), or depression and at least one chronic cardiometabolic condition. The depression data for the measure was derived from the score on the Patient Health Questionnaire, a nine-item (PHQ-9) quick depression assessment used to identify respondents with depression [22,23]. Individuals with scores 10 or higher were considered depressed [23]. The cardiometabolic component of the measure was derived from responses to items asking if respondents had been told by a doctor that they have hypertension, T2DM, or hyperlipidemia. Respondents were assigned to one of three categories, “no disease” were individuals who did not report a chronic cardiometabolic condition or depression, “only chronic disease” were respondents who did not classify as depressed but reported having a chronic cardiometabolic condition, and “chronic disease with depression” were individuals who were classified as depressed and reported having at least one chronic cardiometabolic condition.

#### 2.2.2. Food Insecurity

The primary independent variable for this study was food insecurity. Adult food insecurity was derived from responses to the 10 adult questions of the 18 items on the Food Security Survey Module (FSSM) [24]. Responses were categorized into four levels based on the number of affirmative responses to 10 items (i.e.., full food security, 0 points; marginal food security, 1–2 points; low food security, 3–5 points; and very low food security, 6–10 points) [24].

#### 2.2.3. Other Independent Variables

Weight status was a categorical variable derived from body mass index that is calculated by dividing weight in centimeters by height in meters squared. The specific categories were underweight (BMI < 18.5), normal weight (BMI = 18.5 to 24.9), overweight (BMI = 25.0–29.9), or obese (BMI ≥ 30) [25]. Health behaviors in this study included factors known to be associated with cardiometabolic health. Diet, physical activity, sedentary behavior, and smoking were assessed using self-report. Diet was measured by a single-item measure asking respondents; “How healthy is your diet?” with five categories ranging from “excellent” to “poor”. Physical activity was measured by two items asking respondents; “How much time do you spend doing vigorous-intensity recreational activities on a typical day?” and “How much time do you spend doing moderate-intensity recreational activities on a typical day?”. Physical activity guidelines for Americans suggests 75 min a week for vigorous-intensity or 150 min a week for moderate-intensity activity [26]. Thus, the physical activity variable was categorized into two categories, met the guidelines or did not meet the guidelines. The sedentary lifestyle variable was measured by a single-item measure asking, “How much time do you usually spend sitting on a typical day?”. It was recoded as a dichotomous variable into having a sedentary lifestyle (i.e., sitting for 180 min per day) or not having a sedentary lifestyle. [27]. Health status was represented by two categories, good to excellent and fair or poor. Tobacco use was coded into three categories: never a smoker, former smoker, and current smoker. Usual source of care was operationalized as having a place to go when in need of care, such as a hospital, doctor’s office, etc., or none.

#### 2.2.4. Study Covariates

Study covariates included self-reported demographic data and socioeconomic status. Age was a continuous variable. Sex was a dichotomous variable (i.e., male/female). Race/ethnicity was measured by a categorical variable with categories for individuals identifying themselves as non-Hispanic White, non-Hispanic Black, Hispanic, and Other. Marital status was a categorical variable with respondents assigned to one of three categories based on their selection—single who never married; single who were widowed, separated or divorced; and married or currently living with a partner. Education was modified into three levels (i.e., less than high school, high school graduate/GED or equivalent, and some college or above). Poverty level was determined based upon poverty to income ratio (PIR) and a cutoff point of 1.0 distinguished between poor and rich [28]. Family size was used as a categorical variable based on the number of people reported in the household and categorized as 1 representing a single individual, 2 representing a family of two, 3 representing a family of three, and 4 or more representing a family of 4 or more people.

### 2.3. Statistical Analysis

Sample characteristics were described by multiple t-tests and chi-square tests. All estimates were weighted to adjust for the differential probabilities of sampling and nonresponse, to represent the total civilian, noninstitutionalized U.S. population [29]. Estimates derived from a sample size smaller than the recommended lower limit in the NHANES analytic guidelines were considered unreliable. Unadjusted and adjusted multinomial logistic regression model controlling for age, sex, and race/ethnicity were estimated to evaluate the significant association of social and behavioral factors and multiple chronic conditions status. Results are expressed as odds ratios with 95% confidence intervals. All analyses were performed using SAS-callable SUDAAN, version 13 (Cary, NC, USA) and all estimates and statistical tests were adjusted for the complex NHANES survey design based upon the National Center for Health Statistics (NCHS) recommendation [30].

## 3. Results

Sociodemographic and cardiometabolic characteristics of respondents by household food security status are shown in Table 1. Approximately 15% of the sample reported low to very low food security (food insecurity). The proportion of non-Hispanic Black and Hispanic adults with food insecurity was 2–3 times higher than non-Hispanic White adults. Respondents who were single, including never married and widowed, separated, or divorced, had a higher prevalence of food insecurity than those who were married or living with a partner. Significant differences in percentages were also found by socioeconomic status (SES). The percentage of adults experiencing food insecurity was approximately 30% among those with less than a high school diploma and only 10% among those with some college or above. Concerning poverty level, 37% of respondents who were below the poverty level reported food insecurity compared to 11% of those above the poverty level. While a family size of two seemed to have a protective effect, as family size increased so did the percentage of the sample experiencing food insecurity.

There were significant differences in the percentages of food insecurity reported by weight status. Respondents who reported being underweight had the highest rate of food insecurity at 21%, followed by those who reported obesity at 19%. Food insecurity was 14% among those reporting normal weight and 13% among those reporting overweight. Among those who reported no disease, the percentage of food insecurity was 13%. The percentage of food insecurity was significantly higher at 35% among respondents reporting chronic disease with depression.

Significant differences in food security status were also associated with health lifestyle behaviors, as shown in Table 2. Approximately 60% of respondents reporting fair or poor diets also reported food insecurity. Similarly, respondents reporting fair or poor health reported higher rates of food insecurity. Food insecurity was higher among current smokers when compared to those who stopped smoking or never smoked. Those without a usual source of care reported food insecurity at 25% compared to 14% among those with a usual source of care.

Results from multinomial logistic regression models are presented in Table 3. Several demographic, social, and health lifestyle factors were significantly associated with increased risk of chronic cardiometabolic conditions and mental–physical comorbidities relative to having no disease. With respect to sex, the odds for females were lower than the odds for males with only chronic disease relative to no disease. Non-Hispanic Black adults had greater odds than non-Hispanic White adults with only chronic disease relative to no disease. There were no significant differences found by marital status, education, poverty level, or family size, food security, or healthy diet with only chronic disease relative to having no disease.

For perceived health status, those reporting fair or poor health had higher odds than those reporting good to excellent health with only chronic disease relative to having no disease. Respondents who reported being a current smoker had 1.4 higher odds compared to those who never smoked with only chronic disease relative to having no disease. Additionally, those who reported having a usual source of care had higher odds than those who do not have a usual source of care with only chronic disease relative to no disease.

As it relates to mental–physical comorbidities, the odds for females were 70% greater than the corresponding odds for males with chronic disease and depression relative to no disease. The odds for Hispanic adults were lower than non-Hispanic White adults with mental–physical comorbidities relative to no disease. There were no significant differences found by marital status, education, poverty level, or family size.

Overall, food insecurity was significant for respondents with mental–physical comorbidities when compared to the no disease group. Specifically, the odds for highly food insecure respondents relative to food secure sample members were 3.6 (CI: 2.26–5.76) times greater for having mental–physical comorbidities relative to no disease. Those reporting a poor diet had 4.3 times higher odds than respondents reporting an excellent diet with mental–physical comorbidities relative to no disease. Additionally, the odds for respondents who perceived their health as fair or poor were 6.3 times higher than the odds for those perceiving their health as good to excellent with mental–physical comorbidities relative to no disease.

Behavioral factors such as smoking status including being a current or former smoker and having a usual source of care were both significantly associated with mental–physical comorbidities relative to having no disease. Current smokers had 2.2 higher odds compared to those who never smoked with mental–physical comorbidities relative to no disease. Respondents who reported having a usual source of care had 2.8 higher odds compared to those reporting no usual source of care with mental–physical comorbidities relative to no disease.

## 4. Discussion

Considering the prevalence of chronic disease comorbidities, including co-occurring depression and cardiometabolic comorbidity, among U.S. adults, it is critical to identify risk factors that may influence disease management, progression, and long-term health outcomes. This study provides timely and relevant evidence of significant individual, social, and structural level determinants associated with depression and cardiometabolic comorbidities in U.S. adults. The main finding from this study is that food insecurity is significantly associated with greater odds of mental–physical comorbidities relative to no disease.

This finding corresponds to previous research that found food insecurity to be a risk factor for multiple chronic conditions and for depression, in independent analyses [1,4]. This study confirms the association between food insecurity and co-occurring mental and physical comorbidities in a national sample. These findings suggest that increasing food security should be a target for prevention and management of mental–physical comorbidities prior to or along with lifestyle interventions.

The impact of food insecurity on health outcomes is receiving increased attention. However, most food assistance programs and social support programs focus on low-income families without considering complex clinical status. It is well established that food insecurity is significantly associated with a higher prevalence of depression [1,31] including among those with diabetes [15]. Leung and colleagues [1] found that those without food stamp benefits showed the highest risk of depression. This suggests that additional support including food support and nutrition education, in addition to standard of care, is needed for individuals experiencing food insecurity. Legitimate food support is needed since proper food provision and nutrition counseling can lower healthcare spending and healthcare utilization [32].

These findings also highlight the need for different primary and secondary prevention strategies to effectively address food insecurity amid the rising prevalence of cardiometabolic comorbidities, especially those co-occurring with depression. Characterizing sociodemographic, lifestyle behaviors, and social environmental factors associated with comorbid chronic disease and depression is important for informing and developing targeted intervention research. To our knowledge, this is the first study to examine food insecurity and risk of co-occurring depression and cardiometabolic comorbidities compared to chronic disease and no disease among a representative sample of U.S. adults. This information is critical for improving prevention and self-management, including adherence and self-activation among those experiencing mental–physical comorbidities.

We also found individual and social factors were associated with mental–physical comorbidities. Age and sex were both associated with comorbidity status. Specifically, those with mental–physical comorbidities were more likely to be older and female. Other studies have also found significant correlations between comorbid depression and chronic disease among women [33]. The current study confirms these findings in a national data set.

Social-level factors such as marital status and family size were also associated with comorbidity status. Single individuals, who were never married or who were divorced/widowed had higher rates of mental–physical comorbidities compared to those who were married or living with a partner. However, individuals who were never married had lower rates of chronic disease overall. Future studies should seek to disentangle these relationships to meet the needs of individuals and families.

Structural-level determinants were associated with mental–physical comorbidities together with individual health lifestyle behaviors, self-reported health, and having a usual source of care. Lower socioeconomic status was associated with a higher rate of mental–physical comorbidities. Moreover, among individuals with depression and cardiometabolic comorbidities, self-perception of health status, food security, and healthy diet were significantly lower when compared to the other groups.

The study has some limitations. First, due to limited variables collected by the NCHS, clinical diagnosis of depression is unknown. However, since this study used a standard depression screener to identify depression, it is reliable. We were unable to separate those with Type 1 versus Type 2 diabetes in the analysis; however, only 5% of the sample has Type 1 diabetes. Additionally, as with all cross-sectional databases, causal relationships may not be established. Preliminary models included physical activity and sedentary lifestyle as measures of behavioral risk factors. However, due to small sample size less than 30, these significant values are not valid as population estimates. Subsequently, these two behavior variables were excluded in final models. Finally, participants were asked whether they have a usual source of care that may not account for types of services they received for comorbid conditions, which may present imbalanced health outcomes in individuals who received tailored treatment for multiple conditions despite having a usual source of care. Despite the noted limitations, we are confident in our results as they contribute to the growing body of knowledge surrounding mental–physical comorbidities.

## 5. Conclusions

Food insecurity is significantly associated with mental–physical comorbidities. Understanding the risk factors for and relationship between mental–physical comorbidities may assist in improving health promotion approaches by allowing researchers and practitioners to tailor interventions to the needs of specific subgroups. Health practitioners may consider partnerships with community-based organizations serving high-risk populations to provide prevention and management related nutrition education along with screenings to address mental–physical comorbidities. Future research should consider the role of supplemental social programs designed to address food security, as it appears to be contributing to disparities in chronic disease outcomes.

## Figures and Tables

**Table 1:** Characteristics of study population (%) by food security status using NHANES 2013–2016 data.

	Full Security (*n* = 7863)	Marginal Security (*n* = 1353)	Low Security (*n* = 1275)	Very Low Security (*n* = 963)	*p*-Value
Food Security Status	74.9	9.8	8.6	6.7	
Age * (mean)	48.3	41.1	41.2	40.5	<0.01
Sex					
Male	75.6	9.4	8.2	6.8	NS
Female	74.3	10.1	9.0	6.7	
Race/Ethnicity *					
NH White	81.3	7.3	5.8	5.5	<0.001
NH Black	60.5	15.9	13.0	10.5	
Hispanic	56.4	15.8	18.3	9.6	
Others	77.1	9.6	7.2	6.1	
Marital Status *					
Married, living with partner	79.7	8.2	7.1	5.0	<0.001
Widowed, separated, divorced	69.2	11.2	10.1	9.5	
Never married	67.7	11.5	11.3	9.5	
Education *					
Less than high school	56.6	13.8	18.5	11.2	<0.001
High school graduate/GED or equivalent	68.7	12.1	10.2	9.0	
Some college or above	82.4	7.4	5.3	4.8	
Poverty Level *					
Poor (PIR < 1)	43.7	19.2	18.4	18.8	<0.001
Rich (PIR ≥ 1)	81.5	7.8	6.3	4.5	
Family Size *					
1	73.1	9.6	7.4	9.8	<0.001
2	84.8	6.2	5.3	3.8	
3	73.0	11.3	8.6	7.1	
4 +	68.7	12.1	12.3	7.0	
BMI (kg/m^2^) *					
Underweight	61.3	17.3	12.1	9.3	<0.001
Normal	77.42	9.1	7.3	6.3	
Overweight	78.0	9.1	7.6	5.4	
Obese	70.7	10.6	10.5	8.2	
Comorbidity Status *					
No disease	78.0	9.5	7.3	5.2	<0.001
Only chronic disease	78.5	8.3	7.7	5.6	
Chronic disease with depression	50.8	14.7	14.6	19.9	

* Indicates significance.

**Table 2:** Health lifestyle behaviors by food security status using NHANES 2013–2016 data.

	Full Security (*n* = 7863)	Marginal Security (*n* = 1353)	Low Security (*n* = 1275)	Very Low Security (*n* = 963)	*p*-Value
Healthy Diet *					
Excellent	85.5	4.9	5.1	4.5	<0.001
Very Good	86.23	6.9	5.0	1.8	
Good	77.0	9.9	7.7	5.5	
Fair	63.8	11.6	15.0	9.6	
Poor	51.5	12.7	15.5	20.4	
Health Status *					
Good or above	78.6	9.3	6.9	5.2	<0.001
Fair or poor	59.7	11.5	14.8	14.0	
Tobacco Use *					
Never smoker	78.5	9.0	7.9	4.7	<0.001
Former smoker	81.0	7.6	6.5	4.8	
Current smoker	58.9	13.4	12.9	14.8	
Usual Source of Care *					
Yes	77.5	8.9	8.0	6.0	<0.001
No	61.0	14.1	14.1	10.8	

* Indicates significance.

**Table 3:** Results of multinomial logistic regression for factors associated with mental–physical comorbidity status among U.S. adults using NHANES 2013–2016 data.

Factors	Odds Ratio (95% CI)	
	Only Chronic Disease	Chronic Disease with Depression
Age *	1.1 (1.06–1.08) *	1.1 (1.04–1.09) *
Sex *		
Male	1.00 (Ref)	1.00 (Ref)
Female	0.8 (0.67–0.99) *	1.7 (1.06–2.84) *
Race/Ethnicity *		
NH White	1.00 (Ref)	1.00 (Ref)
NH Black	1.4 (1.10–1.66) *	1.1 (0.72–1.84)
Hispanic	0.8 (0.62–1.12)	0.5 (0.36–0.79) *
Others	1.1 (0.86–1.43)	1.1 (0.60–2.01)
Marital Status		
Married, living with partner	1.00 (Ref)	1.00 (Ref)
Widowed, separated, divorced	1.0 (0.82–1.25)	1.4 (0.87–2.32)
Never married	1.1 (0.75–1.55)	0.9 (0.50–1.68)
Education		
Less than high school	1.00 (Ref)	1.00 (Ref)
High school graduate/GED or equivalent	1.0 (0.82–1.24)	0.7 (0.46–1.03)
Some college or above	1.0 (0.78–1.31)	0.6 (0.35–1.02)
Poverty Level		
Poor (PIR < 1)	1.00 (Ref)	1.00 (Ref)
Rich (PIR ≥ 1)	0.9 (0.75–1.19)	1.0 (0.62–1.46)
Family Size		
1	1.00 (Ref)	1.00 (Ref)
2	1.1 (0.81–1.66)	1.3(0.71–2.43)
3	0.8 (0.61–1.11)	1.2 (0.68–2.11)
4 +	1.0 (0.68–1.35)	1.0 (0.59–1.62)
Food Security *		
Full	1.00 (Ref)	1.00 (Ref)
Marginal	1.2 (0.81–1.66)	2.4 (1.06–5.43) *
Low	1.2 (0.77–1.86)	2.2 (1.23–3.95) *
Very low	1.5 (0.99–2.36)	3.6 (2.29–5.76) *
Healthy Diet *		
Excellent	1.00 (Ref)	1.00 (Ref)
Very Good	0.8 (0.54–1.30)	0.7 (0.28–1.63)
Good	1.2 (0.69–1.95)	1.6(0.81–3.08)
Fair	1.7 (1.00–2.91)	2.8 (1.34–5.85) *
Poor	1.7 (0.87–3.45)	4.3 (1.47–12.59) *
Health Status *		
Good or above	1.00 (Ref)	1.00 (Ref)
Fair or poor	2.0 (1.57–2.56) *	6.3 (4.15–9.71) *
Tobacco Use *		
Never smoker	1.00 (Ref)	1.00 (Ref)
Former smoker	1.4 (1.08–1.70) *	1.6 (1.04–2.38) *
Current smoker	1.4 (1.04–1.92) *	2.2 (1.46–3.47) *
Usual Source of Care *		
No	1.00 (Ref)	1.00 (Ref)
Yes	1.6 (1.19–2.08) *	2.8 (1.56–4.84) *

* *p* < 0.05.

## Data Availability

The National Health and Nutrition Examination Survey data are publicly available from the Centers for Disease Control and Prevention.
